# A Case of Severe Trigeminal Neuralgia Successfully Treated by Excision of the Gasserian Ganglion

**Published:** 1908-09

**Authors:** Ernest W. Hey Groves

**Affiliations:** Assistant Surgeon to the Bristol General Hospital


### A CASE OF SEVERE TRIGEMINAL NEURALGIA SUCCESSFULLY TREATED BY EXCISION OF THE GASSERIAN GANGLION.

BY

Ernest W. Hey Groves, M.S., F.R.C.S.,
*Assistant Surgeon to the Bristol General Hospital.*

Fortunately, trigeminal neuralgia of severe degree is a rare disease, and its radical treatment has been one of the recent notable advances of surgery. It may be of interest, therefore, to describe the symptoms and operative details in a case of exceptional severity.

L.R., a married woman of 40, was sent to me by Dr. Thomas, of Newport, in February, 1907, after she had been attending as an out-patient at the hospital there for five weeks. She had had salicylates, aspirin, gelsemium, and other drugs by mouth, together with various local applications, but none of these had the slightest permanent effect upon the pain. Her history was that she had had seven children, but no miscarriages or any other illness. About five years previously she had a bad attack of neuralgia on the right side of her face, which lasted on and off for about six months. Thirteen teeth were extracted without much benefit, and this left her edentulous on the right side.

Ten months prior to my seeing her the present attack began, after she had been suckling a child for twelve months. During the last two months of this period the pain had become much more constant and the paroxysms more severe. She was a thin, short woman, of quiet and placid disposition, quite free from any obvious neurotic tendencies. The pain, which was strictly limited to the right side of the face, was of a constant and dull character, affecting the cheek, jaws and tongue. Every few minutes a violent paroxysm began in the chin and spread up to the forehead, and lasted from one-half to five minutes. During the time that she was being examined, it was rare for as much as five minutes to elapse without such a spasm occurring; but when she was left to herself she liked to draw the curtains round her bed to exclude any possible draught, but even then the attacks recurred every twenty minutes to half an hour during both day and night. She described the pain as being like fire or red-hot wires being driven into hollow teeth. The skin of the face was rough and cracked on the right side, and of a darker colour than that on the opposite. The hair was quite lost from the right temple and eyebrow as the result of the franctic rubbing to which she subjected herself during each paroxysm. No spots of anæsthesia were observed. Mr. Walker kindly examined her eyes, and found the discs healthy, but a high degree of hypermetropic astigmatism was present on both sides, amounting to 6.5 D in the vertical diameter and 9 D in the horizontal. Treatment by atropine had no effect upon the pain. The heart, lungs, and kidneys were healthy. After a consultation with Dr. Michell Clarke, it was decided that it was quite useless to try any further palliative measures, and the excision of the Gasserian ganglion was decided on. On February 27th, 1907, the operation was performed. Following Crile's suggestion with a view to lessening the hemorrhage, the right common carotid artery was exposed by the usual incision, and a temporary ligature placed upon it. The right eyelids were stitched together to avoid post-operative neuropathic keratitis. A ∩ shaped flap of skin and muscle was turned down in the right temporal region, the base of the flap being over the zygoma. A piece of bone was removed by a one-inch trephine, and the opening enlarged, especially downwards, by Hoffman's forceps. On separating the dura the bleeding was so great as to quite obscure all view of the cavity in the skull. A constant suction apparatus, arranged on the principle of a Cathcart's irrigator, was employed with excellent results to keep the blood out of the cavity. The nozzle of this had to be changed from time to time owing to the rapid clotting of blood. The trunk of the middle meningeal artery on the deep surface of the dura served as a guide to the foramen ovale, through which the third division of the fifth nerve leaves the skull. The artery was cut without any bleeding, and this appeared to be the sole gain derived from the ligature of the carotid. At this stage the third division of the nerve was seen running downwards and the second forwards, and the ganglion at their junction ; but owing to the way in which the dura mater splits to enclose the nerves and cavernous sinus their definition was anything but clear, especially as their situation is nearly two inches deep from the surface. Both divisions were cut ; but whilst an attempt was being made to free the ganglion a sudden furious bleeding occurred, similar to that which follows the opening of the lateral sinus in mastoid operations, and this made it necessary to plug the wound and temporarily to abandon the operation. The ligature on the carotid was replaced by a permanent one, lest its removal should restart the bleeding. The operation was followed by prolonged unconsciousness lasting $7\frac{1}{2}$ hours, and accompanied by slow breathing and a pulse of 40. During the succeeding days her condition gave rise to no anxiety, and she was free from pain, saying that the side of her face and tongue felt dead. On March 4th, 1907, the operation was completed, and this stage proved so easy that I should be inclined on future occasions, instead of clamping the carotid, to deliberately operate *a deux temps.* The root of the nerve was isolated and cut, and the ganglion twisted out by pressure forceps. A piece of iodoform gauze was left in the depths of the wound and brought out between the posterior edges of the skin incision, which otherwise was sewn together. On this occasion there was practically no shock, and the patient made a rapid and uneventful recovery. When I showed the patient before the Bristol Medico-Chirurgical Society last March her condition was as follows : The hair had grown well over the scar, and also over the temple and eyebrow, and she was perfectly free from any pain or discomfort. Over the right cheek and lips there was an area of anæsthesia to light touch (epicritic sensation), which ended in an abrupt right angle at the tragus of the ear. There was no alteration of sensation in the eyelids or forehead ; and corneal sensation was dulled but not absent ; evidently the ophthalmic division of the nerve had partly escaped removal. Within the anæsthetic area she could still feel deep pressure or the prick of a pin (protopathic sensation). The external auditory meatus and tympanic membrane were normally sensitive. The inner surface of the cheek, the gums and tongue in its right half, were both anæsthetic and analgesic, and she was quite unaware of a needle being thrust through the latter. The area of anæsthesia ended behind the anterior faucial pillar. It is probable, from the analogy of other cases, that these areas of anæsthesia are quite permanent.

In view of the extreme misery to which patients are reduced who suffer from trigeminal neuralgia, which is often so great as to induce them to resort either to suicide or to the worst form of morphia habit, no operation which produces a radical cure can be regarded as too severe; and considering that more than ten years have now elapsed since excision of the Gasserian ganglion was performed, and that none of the patients have had any return of the symptoms when the operation has been complete, it may be fairly claimed that this procedure is a radical cure.

All such measures as neurectomy of the branches of the nerve, the injection of osmic acid or absolute alcohol into the nerve foramina, are merely of temporary effect, which seldom lasts longer than a few months.

As regards the method of the removal of the ganglion, the consensus of opinion is favourable to the temporal route, the alternative being the zygomatic route, in which the zygoma is turned down, the coronoid process of the jaw is turned up, and the skull is opened from below by chiselling round the foramina through which the nerves leave the skull. This latter method is more difficult, and the ganglion can only be extracted piecemeal with great uncertainty.

It has also been suggested that the root of the ganglion can be cut in the posterior fossa of the skull, but the technical difficulties of this operation are greater than those of the more radical operation upon the ganglion.

In conclusion, I might add that the excised ganglion was examined by Dr. Michell Clarke (to whom I am greatly indebted for his kindness and help over this case), who cut sections of it, and stained them by various methods. These sections, which were exhibited at the same time as the patient, showed no recognisable pathological change, and this is in accordance with the findings of other observers. Nothing is more remarkable in this disease than the absolute obscurity of its origin as compared with the definite character of its symptoms and treatment.

